# MS-PANet: Multi-Scale Spatial Pyramid Attention for Effective Drainage Pipeline Image Dehazing

**DOI:** 10.3390/jimaging12050189

**Published:** 2026-04-27

**Authors:** Ce Li, Xinyi Duan, Zhongbo Jiang, Yijing Ding, Quanzhi Li, Zhengyan Tang, Feng Yang

**Affiliations:** 1 School of Artificial Intelligence, China University of Mining and Technology-Beijing, Beijing 100083, China; celi@cumtb.edu.cn (C.L.);; 2 Key Laboratory of Safety Detection and Evaluation of Urban Roads and Underground Space, Ministry of Emergency Management, Beijing 100083, China; 3 Aerospace Information Research Institute, Chinese Academy of Sciences, Beijing 100094, China; 4 School of Geosciences and Surveying Engineering, China University of Mining and Technology-Beijing, Beijing 100083, China

**Keywords:** drainage pipeline, image dehazing, multi-scale, pyramid attention mechanism, dense feature fusion

## Abstract

Urban drainage pipelines are crucial for flood control, drainage, and environmental quality. However, fog within pipelines degrades image quality, hindering the identification of damage features such as cracks and leaks. Existing dehazing algorithms struggle with the unique challenges presented by drainage pipelines, such as their cylindrical structure, non-uniform lighting, and multi-scale particulate interference, leading to inadequate feature extraction and weak cross-channel dependency modeling. To address these issues, we propose a novel drainage pipeline image dehazing network based on a pyramid attention mechanism. Specifically, our proposed method incorporates a custom-designed multi-scale spatial pyramid attention (MSPA) module, which combines hierarchical pyramid convolution and spatial pyramid recalibration modules. This enables the dynamic adjustment of multi-scale feature weights and the effective modeling of cross-channel long-range dependencies. Extensive experiments demonstrate that our network achieves superior dehazing performance across diverse underground environments, particularly in synthetic foggy dataset under real pipeline conditions, outperforming state-of-the-art dehazing algorithms. This proposed approach provides a reliable solution for high-precision visual inspection in complex pipeline scenarios.

## 1. Introduction

In the contemporary era of accelerated urbanization, urban drainage systems have emerged as pivotal infrastructure, assuming critical responsibilities in stormwater management, pollution abatement, and ecological preservation. As the indispensable cyber–physical infrastructure for smart city ecosystems [1], these underground lifelines not only facilitate the efficient conveyance of rainwater and wastewater but also serve as essential safeguards against flooding, directly influencing urban safety and public well-being. The operational integrity of drainage pipelines is inextricably linked to urban quality of life, economic vitality, and environmental sustainability.

Recent advances in computational imaging have established physics-aware dehazing as a promising solution for subsurface vision systems [2]. Modern deep dehazing architectures [3] typically employ multi-scale feature fusion with attention mechanisms [4], achieving significant PSNR improvements on natural scene benchmarks [5]. However, their direct application to pipeline inspection encounters fundamental challenges, such as cylindrical geometry-induced perspective distortions, non-uniform illumination from point light sources, and multi-modal scattering from polydisperse aerosols [6]. Consequently, existing dehazing methods exhibit limited performance due to inadequate modeling of anisotropic scattering kernels and insufficient cross-channel dependency learning [7].

To address these limitations, this study introduces a novel dehazing framework named MS-PANet, which integrates the multi-scale boosted dehazing network with dense feature fusion (MSBDN) architecture [8] with a multi-scale spatial pyramid attention (MSPA) mechanism. The main contributions of this study are summarized as follows:

(a) We propose a novel multi-scale spatial pyramid attention (MSPA) module, which enhances multi-scale spatial feature extraction through structural regularization and efficiently constructs long-range channel dependencies, thereby enriching feature representation for subsequent analysis.
(b) By incorporating the MSPA module into ResNet’s residual blocks using 3 × 3 convolutions, we develop a novel dehazing framework, MS-PANet, that integrates the Multi-Scale Boosted Dehazing Network with Dense Feature Fusion backbone. This framework enables adaptive channel attention recalibration while capturing comprehensive multi-scale feature hierarchies.
(c) A stereo depth camera-equipped pipeline inspection robot was deployed to acquire clear pipeline imagery and corresponding depth maps. Utilizing these data, we generated synthetic hazy images via atmospheric scattering models and collected hazy images in real-world environments, culminating in the creation of the CDPD-55000 dataset—a valuable resource for dehazing algorithm development and evaluation.
(d) Extensive experiments on the CDPD-55000 dataset demonstrate that our method achieves state-of-the-art performance in drainage pipeline dehazing scenarios, significantly outperforming contemporary algorithms across multiple evaluation metrics. These results underscore the practical utility of our approach in enhancing the intelligence and reliability of urban drainage pipeline inspection systems.

## 2. Related Work

### 2.1. Pipeline Inspections

Early monitoring of drainage pipes mainly relied on manual inspection, acoustic detection, gas detection, and other indirect means [9]. These methods generally yield non-intuitive data, suffer from imprecise positioning, and rely heavily on empirical judgment; consequently, they fail to meet the demands of modern cities for efficient pipeline operation and maintenance. closed-circuit television (CCTV) inspection [10] is the most common pipeline defect detection method. The core of the CCTV approach involves deploying robots or crawlers equipped with high-definition cameras and lighting systems into the pipeline to collect and transmit image data in real time, ultimately locating and recording defects in conjunction with a geographic information system (GIS). Its intuitiveness, accuracy, and data traceability not only improve inspection efficiency but also establish it as an indispensable technical pillar in smart city infrastructure management [11,12].

### 2.2. Dehazing Algorithms

In the field of image dehazing, existing algorithms are primarily divided into two categories: those based on physical models and those based on deep learning. Physical model-based methods typically simulate the hazy effect of images using the atmospheric scattering model (ASM) [13] and restore image clarity by reversing this process. Algorithms in this class, such as dark channel prior (DCP) [14] and color attenuation prior (CAP) [15], are interpretable but may result in distortions in extreme scenes, such as heavy haze or complex illumination. Conversely, deep learning-based methods [16,17] train models to learn the complex mapping relationship from hazy to clear images. These methods have become a research hotspot, driving significant progress and applications in image processing and computer vision. The traditional physical model-based dehazing image restoration method relies on manually designed physical priors derived from atmospheric scattering models. These methods have interpretability and computational efficiency, but they often fail due to the presence of anisotropic scattering in pipeline environments, non-uniform illumination caused by point light sources, and multi-scale particle interference. In contrast, deep learning based methods directly learn the mapping from foggy images to clear images from data, which can implicitly model these complex degradation processes without explicitly describing the physical model.

### 2.3. Multi-Scale Feature Extraction

Multi-scale feature extraction is a core strategy for improving the performance of visual recognition tasks, and the key lies in capturing visual patterns at different granularities through hierarchical modeling. Convolutional neural networks (CNNs) naturally facilitate a coarse-to-fine feature characterization process through a layer-by-layer stacked local perception mechanism. To further enhance this property, researchers have proposed various architectural improvements. Multi-branch convolution-based architectures achieve multi-scale information fusion through the cooperative operation of differentiated filters. Notably, the InceptionNet series [18] lays an important foundation for subsequent research by designing parallel convolution paths at different scales. These methods have shown significant application value in several fields, such as medical image analysis [19], text processing [20], and geological exploration [21].

### 2.4. Attention Mechanisms

Attention mechanisms have been widely integrated into CNN architectures to enhance the context-awareness of models by dynamically assigning feature weights to strengthen the representation of key information. Existing studies have mainly used two paradigms to integrate attention mechanisms into convolutional neural networks: one is to construct independent attention modules to model the correlation between features, and the other is to replace specific structural components of CNNs to enhance local feature interactions. Regarding independent attention modules, SENet [22] pioneered the end-to-end channel attention training paradigm with its proposed squeeze-excitation mechanism that establishes channel dependencies through global average pooling and fully connected layers. Subsequent studies have optimized different components of the framework: SPANet [23] introduces spatial pyramid pooling in the squeeze module to obtain multi-scale contextual information; ECA [24] replaces the fully-connected layer with 1D convolution to significantly reduce the computational complexity while maintaining performance. RIDCP [17] leverages high-quality codebook priors encapsulated in a discrete latent space to effectively restore fine details in real-world hazy images. DehazeFormer [16] adapts the Vision Transformer architecture with modified normalization and activation functions to enhance feature representation for single image dehazing.

### 2.5. Discussion

Although existing image dehazing algorithms have made significant progress in natural scenes, their application to drainage pipelines remains challenging. When confronted with the unique cylindrical spatial structure, non-uniform lighting conditions, and multi-scale suspended particle interference, these methods suffer from insufficient feature extraction granularity and weak modeling of cross-channel dependencies, ultimately limiting dehazing performance. In this paper, we propose a novel framework that combines the MSBDN architecture with a multi-scale spatial pyramid attention mechanism. More specifically, a novel multi-scale spatial pyramid attention (MSPA) module is proposed to extract multi-scale spatial features more effectively. By leveraging structural regularization and spatial information, this module achieves superior feature representation while efficiently constructing long-range channel dependencies.

## 3. Methodology

This section systematically describes the architectural design of the MSPA module and the final construction of the novel backbone network, MS-PANet. Section 3.1 introduces the overall architecture of MS-PANet, while Section 3.2, Section 3.3 and Section 3.4 elaborate on the hierarchical computational process of the MSPA module and its core components, the HPC and SPR modules, respectively.

### 3.1. Overall Framework

Existing research indicates that utilizing multi-scale information from images can significantly improve model performance. Building upon this premise, we propose a novel architecture that integrates the MSBDN framework with a multi-scale spatial pyramid attention (MSPA) mechanism. This integration fully exploits multi-scale image and feature information for effective dehazing, thereby facilitating subsequent advanced visual detection tasks using networks such as YOLO [11,12,25,26,27].

**Figure 1:**
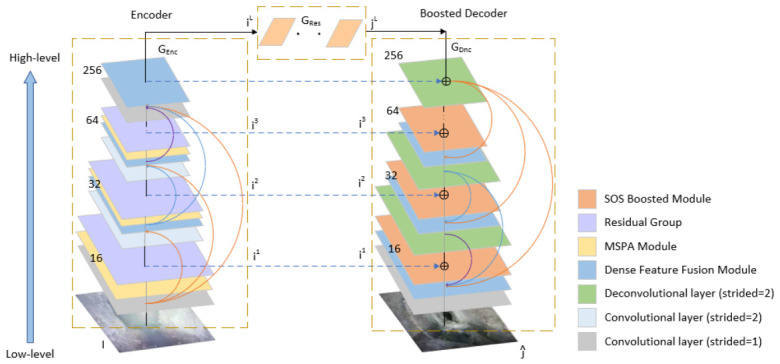
Overall framework of MS-PANet.

As illustrated in Figure 1, the proposed method comprises three primary components: an encoder module, an enhanced decoder module, and a feature recovery module. The encoder module focuses on extracting discriminative feature representations from the input image, progressively reducing spatial dimensions through successive convolutional layers to capture high-level semantic features. The enhanced decoder module utilizes skip connections from the MSBDN structure to aggregate the encoder’s output feature maps with the decoder’s input representations. Within the decoder, a multi-scale feature fusion module is employed to restore fine-grained image details and enhance contrast. To optimally manage spatial details and contextual information, we introduce the MSPA mechanism into the encoder, replacing traditional convolutions. This mechanism adaptively captures multi-scale image features.

### 3.2. Multi-Scale Spatial Pyramid Attention

**Figure 2:**
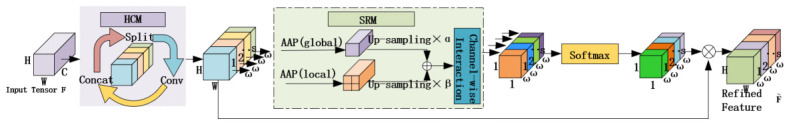
Our proposed MSPA module.

To construct a scalable channel attention mechanism, this study proposes the MSPA module, whose overall framework is illustrated in Figure 2. Its innovation lies in the synergistic optimization of multi-scale spatial feature extraction and cross-channel dependency modeling. It consists of three core components: the hierarchical pyramid convolution (HPC) module, the spatial pyramid recalibration (SPR) module, and the Softmax operation. Specifically, the HPC module extracts multi-scale spatial information; the SPR module learns channel attention weights to build cross-dimensional interactions; and the Softmax operation recalibrates these weights to establish long-range channel dependencies. This module operates through a four-stage computational process, as follows.

**Stage 1: Multi-scale Feature Extraction Using HPC**

Given an input feature map $F\in R^{C\times H\times W}$ (where *C* denotes the number of channels, and *H* and *W* represent spatial dimensions), the process begins by enhancing multi-scale features through the hierarchical pyramid convolution (HPC) module. This process can be formalized as:

(1) $$\hat{F}=HPC\left(F\right),$$

where $\hat{F}\in R^{C\times H\times W}$ is the multi-scale feature group generated by HPC(·) and contains *s* spatially aware enhanced sub-features. More specific calculations in HPC are detailed in Section 3.3).

**Stage 2: Channel Relationship Modeling Using SPR**

The features at each scale ${\hat{F}}_{i}\in \hat{F}$ are input into the spatial pyramid recalibration (SPR) module to efficiently learn channel attention. For the *i*-th enhanced feature map subset ${\hat{F}}_{i}$ (where i∈{1,2,…,s}), the corresponding channel weights $V_{i}$ are computed and concated as the channel weights $V\in R^{C\times 1\times 1}$. The process in the SPR module is formulated in Equation (2) and will be illustrated in Section 3.4.

(2) $$V=SPR(\hat{F}).$$

**Stage 3: Cross-Scale Dependency Construction Using Softmax**

The channel weights at each scale $V_{i}\in V$ are input into the softmax function to recalibrate attention as

(3) $$A_{i}=Softmax\left(V_{i}\right)=\frac{exp\left(V_{i}\right)}{\sum_{i=1}^{s}exp\left(V_{i}\right)}.$$

Through this approach, long-range channel dependencies between different feature map subsets are established. A denote the entire recalibrated channel weights generated in a concatenated manner as

(4) $$A=Concat\left(\left[A_{1},A_{2},\ldots ,A_{s}\right]\right).$$

**Stage 4: Feature Fusion and Output**

Finally, the *i*-th enhanced feature map subset is multiplied by its corresponding attention weight $A_{i}$ to obtain the refined output feature map. This process can be written as follows:

(5) $${\tilde{F}}_{i}=A_{i}⊗{\hat{F}}_{i}.$$

In Equation (5), the symbol ⊗ denotes element-wise multiplication. The MSPA mechanism in our framework significantly enhances the discriminative power and spatial awareness of features by cascading multi-scale attention information. Similarly, the entire refined output feature map is computed as follows:

(6) $$\tilde{F}=Concat([{\tilde{F}}_{1},{\tilde{F}}_{2},\ldots ,{\tilde{F}}_{s}]).$$

Based on the above description, our MSPA effectively integrates multi-scale spatial information and cross-channel attention into a whole module, which helps capture more discriminative features and enhance multi-scale representation capabilities. Subsequently, we comprehensively validate the superiority of this method, with detailed experimental results and analysis provided in Section 4.

### 3.3. Hierarchical Pyramid Convolution Module

**Figure 3:**
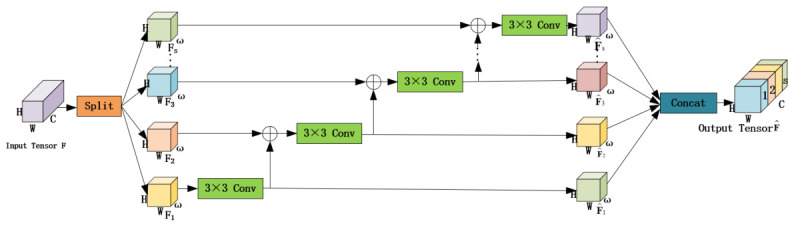
Hierarchical pyramid convolution module.

To address the limitations of existing methods in fine-grained multi-level modeling, we propose the hierarchical pyramid convolution (HPC) module as shown in Figure 3. First, the input feature map F is evenly divided along the channel dimension into *s* sub-features, denoted as $\left\{F_{1},\ldots ,F_{s}\right\}$, where each sub-feature $F_{i}\in R^{\omega \times H\times W}$ satisfies C=s×ω. Then, each $F_{i}$ has a corresponding set of convolutional operators, specifically a 3×3 standard convolution followed by batch normalization, denoted as $\tau_{i}\left(\cdot \right)$. The enhanced output feature subset of $\tau_{i}\left(\cdot \right)$ is denoted as ${\hat{F}}_{i}\in R^{\omega \times H\times W}$.

The Conv operators from different groups are hierarchically connected in a residual-like manner to increase the number of scales that the output features can represent. Structurally, the first group of Conv operators extracts features from $F_{1}$ to generate ${\hat{F}}_{1}$. Then, the feature map subset $F_{i}$ where 1<i≤s is added to the output and fed into $\tau_{i}\left(\cdot \right)$ to obtain ${\hat{F}}_{i}$. This process is repeated multiple times until all input feature splits are processed. This computational process is expressed as follows:

(7) $${\hat{F}}_{i}=\left\{\begin{array}{cc} \tau_{i}\left(F_{i}\right), & i=1\\ \tau_{i}(F_{i}⊕{\hat{F}}_{i-1}), & 1<i\leq s \end{array}\right.$$

In the equation, ⊕ denotes the element-wise summation operation. Finally, the entire enhanced multi-level feature map $\hat{F}\in R^{C\times H\times W}$ can be obtained through concatenation by the following function:

(8) $$\hat{F}=Concat\left(\left[{\hat{F}}_{1},{\hat{F}}_{2},\ldots ,{\hat{F}}_{s}\right]\right).$$

Each group of Conv operators $\tau_{i}\left(\cdot \right)$ can extract feature information from all preceding feature map subsets $\left\{F_{j},j\leq i\right\}$ in the HPC module. Each subset undergoes a 3×3 convolution operation, and the output results have a larger receptive field. Therefore, due to the combinatorial explosion effect, the output of the HPC module $\hat{F}$ contains various combinations and different numbers of receptive field scales.

### 3.4. Spatial Pyramid Recalibration Module

**Figure 4:**
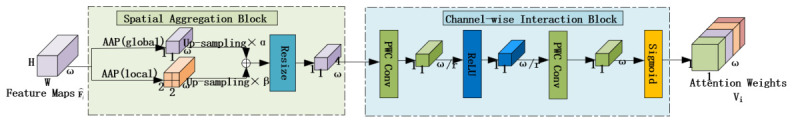
Spatial Pyramid Recalibration Module.

To address the limitations of the HPC module in channel relationship modeling, we design the spatial pyramid recalibration (SPR) module following HPC. As shown in Figure 4, the SPR module consists of a spatial pyramid aggregation (SPA) block and a channel interaction (CI) block. The SPA block adaptively pools the input feature map into two scale channel descriptors. The first descriptor is obtained using the traditional global average pooling (GAP), which has strong structural regularization (i.e., 1×1 average pooling) to generate a global channel descriptor. The second descriptor is generated using local average pooling (LAP) (i.e., 2×2 average pooling), which captures richer feature representations and structural information. Then, the channel descriptors are up-sampled to match the spatial shape of the local channel descriptor and fused via weighted summation. Therefore, based on the above notation, for the enhanced feature map subset ${\hat{F}}_{i}\in R^{\omega \times H\times W}$ as input, the corresponding channel descriptor $Z_{i}\in R^{4\omega \times 1\times 1}$ generated by the SRM module is expressed as follows:

(9) $$Z_{i}=SPA\left({\hat{F}}_{i}\right)=\psi_{re}\left(\alpha ⊗T_{up}\left(P\left({\hat{F}}_{i},1\right)\right)⊕\beta ⊗T_{up}\left(P\left({\hat{F}}_{i},2\right)\right)\right).$$

In the equation, SPA(·) represents the SPA block, P(·,·) denotes the adaptive average pooling layer, $T_{up}\left(\cdot \right)$ refers to the up-sampling function, and $\psi_{re}\left(\cdot \right)$ is the operation that scales a tensor into a vector. Additionally, α and fi are two learnable floating-point parameters optimized through Stochastic Gradient Descent (SGD). By adaptively combining the channel descriptors of two different scales, we effectively integrate the structural regularization and structural information in the SPR, significantly improving the feature representation.

The CI block is designed to solve the problem of modeling the inter-channel relationship for the SPA output descriptor Z. As shown in Figure 4, we encode $Z_{i}$ using a sequence of two 1×1 convolution layers and a Sigmoid function to obtain the corresponding channel attention weight $V_{i}$. This process is formulated as follows:

(10) $$V_{i}=CI\left(Z_{i}\right)=\sigma (f_{2}^{1\times 1}(ReLU(f_{1}^{1\times 1}\left(Z_{i}\right)))).$$

Here, CI(·) denotes the CI block, ReLU(·) is the rectified linear unit activation function, and σ(·) is the sigmoid activation function responsible for mapping the output to the range of (0−1). $f_{1}^{1\times 1}\left(\cdot \right)$ and $f_{2}^{1\times 1}\left(\cdot \right)$ represent the 1×1 convolution operations with parameter matrices (4ω,ω/r) and (ω/r,ω), respectively, where *r* denotes the reduction ratio, primarily used to control the computational cost through dimensionality reduction.

Finally, the attention weights $V\in R^{C\times 1\times 1}$ are the output of SPR module through concatenation along the channel dimension as the following function.

(11) $$V=Concat\left(\left[V_{1},V_{2},\ldots ,V_{s}\right]\right).\;$$

Note that these attention weights for the entire channel are obtained via SPR to facilitate attention interaction without disrupting the original attention weights.

## 4. Experiments

### 4.1. Drainage Pipeline Dataset

**Figure 5:**
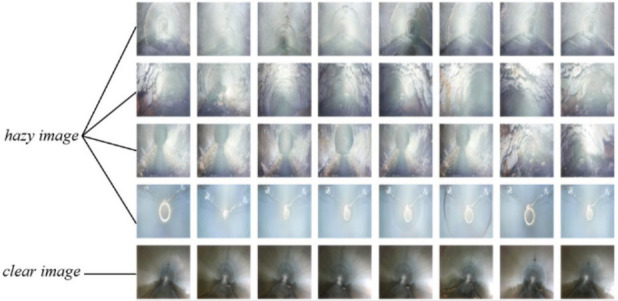
Drainage pipeline dehazing dataset.

During the data collection process, a pipeline inspection robot equipped with a stereo depth camera collected field data from five distinct regions in Beijing. We randomly divided these 5750 clear images into 5500 for training and 250 for testing. Following our work in [28], we set the global atmospheric light value to 1.0 and randomly selecting scattering coefficients in the range of 0.6 to 1.5. We synthesized 10 hazy images for each clear image. This methodology yielded a comprehensive dataset comprising clear pipeline images and their corresponding hazy counterparts, with each clear image augmented by ten different scattering coefficients. Using this approach, we generated hazy images from 5750 clear images collected by the pipeline robot and resized them to 448 × 448 pixels. Of these, 55,000 pairs of hazy and clear images were allocated for training purposes, while 2500 pairs were designated for testing. Finally, we constructed the CDPD-55000 drainage pipeline dehazing dataset, as shown in Figure 5. The following experiments were conducted on the CDPD-55000 dataset.

### 4.2. Ablation Studies

#### 4.2.1. Importance of HPC Module, SPR Module, and Softmax Operation

In this section, we employ MSBDN-RDFF [8] as the baseline model and conduct several ablation experiments to evaluate the practical efficacy of each core component in the proposed MSPA module, including the HPC module, SPR module, and Softmax operation, thereby validating our architectural design. The corresponding experimental results are presented in Table 1. Specifically, the “HPC” configuration denotes replacing the 3×3 convolutional layers with the HPC module in the bottleneck residual blocks of the ResNet while keeping other configurations unchanged. The “SPR” configuration refers to the scale dimension (*s*) in the HPC module being set to 1, which effectively bypasses the hierarchical pyramid structure. The “HPC + SPR (no Softmax)” is equipped with both HPC and SPR modules while removing the Softmax operation.

The “HPC + SPR (Softmax)” configuration corresponds to our proposed method. We can derive the following findings from the experimental results reported in Table 1. These results objectively indicate that the HPC and SPR modules are beneficial for improving dehazing performance. Intuitively, the reason for the above phenomenon is that the HPC module effectively enhances the multi-scale representation capability of the network, while the SPR module efficiently models inter-channel correlations by aggregating more informative channel descriptors. Furthermore, the “HPC + SPR (no Softmax)” configuration further improves restoration quality, proving that the HPC and SPR modules are compatible and their efficacy is complementary, i.e., embedding multi-scale feature representations in channel attention learning is advantageous. Finally, it is worth emphasizing that our approach attains the best result highlighted in Table 1. It is noteworthy that the significant improvement in PSNR (approximately 5.35 dB) from ’HPC + SPR (without Softmax)’ to ’HPC + SPR (with Softmax)’ originates from the cross-scale competition enforced by Softmax. This forces the network to selectively focus on the most informative scales, rather than distributing attention evenly across all scales. This sparse selection is crucial for handling multi-scale fog particles in pipeline images. As a brief conclusion, these empirical results strongly support our design concept and validate the effectiveness of our design solution.

#### 4.2.2. Impact of Scale (*S*) and Channel (ω) Parameters

To investigate the impact of different scale and channel dimensions within the HPC module on our MS-PANet performance, we conducted extensive ablation experiments on the drainage pipeline dataset using MSBDN-RDFF [8] as the baseline. The corresponding results are summarized in Table 2 and Table 3. The parameters *s* and ω denote the number of scales and the number of channels (i.e., filter width), respectively.

First, we evaluate the performance variations induced by different scale settings. As shown in Table 2, we conducted this ablation study by fixing the channel number to 30 (ω=30) and varying the scale dimension *s* from 2 to 5. The experimental results demonstrate that MSPA with various scales consistently outperforms the baseline model in terms of dehazing performance. Interestingly, our MS-PANet with the s=2 configuration achieves a PSNR of 38.27 dB, which is 4.67 dB higher than the baseline. These results fully demonstrate that enhancing multi-scale representation is crucial for improving network performance. It is worth noting that while computational overhead increases sharply with larger scales, the performance does not improve monotonically, but rather reaches an optimal value at s=3. These findings validate the rationality of our parameter selection within the HPC module.

Next, we compare and analyze the impact of different channel dimensions on model performance. As presented in Table 3, we set the scale *s* to 3 and evaluate five different channel dimensions (ω∈{28, 30, 32, 34, 36}) to better observe performance variations. The following conclusions can be drawn from the results: evidently, MS-PANet configurations with different channel dimensions consistently achieve significant PSNR improvements over the baseline. In particular, compared to the original MSBDN-RDFF, the MS-PANet with the ω=28 configuration yields a PSNR improvement of 4.61 dB. These results indicate that augmenting multi-scale feature representation enhances network performance. However, increasing the number of channels leads to a substantial rise in model overhead, while the growth in restoration quality levels off or even saturates. For instance, the ω=30 MS-PANet achieves a 1.11 dB higher PSNR than the ω=28 version. In contrast, the ω=36 MS-PANet obtains a marginal PSNR improvement of only 0.05 dB over the ω=30 version. These empirical findings suggest that the parameter configuration in the HPC module strikes a favorable balance between performance and computational overhead, further validating the correctness and effectiveness of our proposed design.

### 4.3. Comparative Experiments

To validate the engineering applicability of our proposed MS-PANet algorithm, comparative experiments were conducted using the proprietary drainage pipeline-specific dataset (CDPD-55000). Peak signal-to-noise ratio (PSNR) [29] and structural similarity index (SSIM) [30] were adopted as the quantitative evaluation metrics. Higher PSNR values (measured in dB) indicate greater fidelity to the original clear image with minimal distortion, while SSIM values (ranging from 0 to 1) measure structural, luminance, and contrast similarities. Specifically, we compared the dehazing performance of gUNet [31], MIMO-UNet [32], MIMO-UNet++ [32], and MSBDN-RDFF [8] against our proposed MS-PANet on the test dataset.

As shown in Table 4, incorporating the multi-scale spatial pyramid attention mechanism significantly improves network performance. Compared to MSBDN-RDFF, our method achieves a 5.67 dB improvement in PSNR. MixDehazeNet [31] achieves a PSNR of 35.16 dB and SSIM of 0.906, which outperforms MSBDN-RDFF but still falls short of our MS-PANet by 4.16 dB in PSNR. This demonstrates that while MixDehazeNet’s mixed structure block offers some benefit, our proposed MSPA mechanism provides superior multi-scale feature extraction and cross-channel dependency modeling. The hierarchical pyramid convolution module helps the model better capture both global and local features in the image.

The spatial pyramid attention mechanism enables the model to focus on important regions in the image while suppressing irrelevant or less important areas. In the image dehazing task, some areas may have heavier fog and significant detail loss, while other regions may be less affected. By introducing spatial attention, the model can more intelligently handle these regions differently, enhancing its ability to recover details. In the pyramid structure, features at different scales are transmitted progressively, allowing global information at higher layers and local details at lower layers to complement and optimize each other.

Regarding computational complexity, Table 4 reveals that integrating the MSPA mechanism into MSBDN-RDFF increases the model’s parameter count to 52.8 M. However, this trade-off is justified by the substantial performance gains. The proposed mechanism enhances the network’s representational capacity, enabling precise localization of crucial regions and multi-scale feature capture. Therefore, despite the increased computational cost, MS-PANet demonstrates superior potential for practical applications requiring high-precision pipeline inspection.

This phenomenon suggests that although increasing the number of parameters may bring a computational burden, by introducing the multi-scale spatial pyramid attention mechanism, MS-PANet is able to better capture the multi-scale information and spatial features when dealing with complex tasks, which improves the performance of the model. This mechanism can effectively enhance the representation ability of the network, especially when dealing with multi-scale features, which enables the model to locate important regions and information more accurately. Therefore, despite its increased number of parameters, it optimizes the performance to a certain extent and shows its superiority in complex tasks.

To summarize, although the increase in the number of parameters may lead to higher computational costs, the model’s performance can be effectively enhanced through reasonable model design and mechanism innovation, especially showing stronger capability in handling complex tasks. Therefore, the method of MS-PANet has a greater potential in practical applications, especially in fields that require higher accuracy.

**Figure 6:**
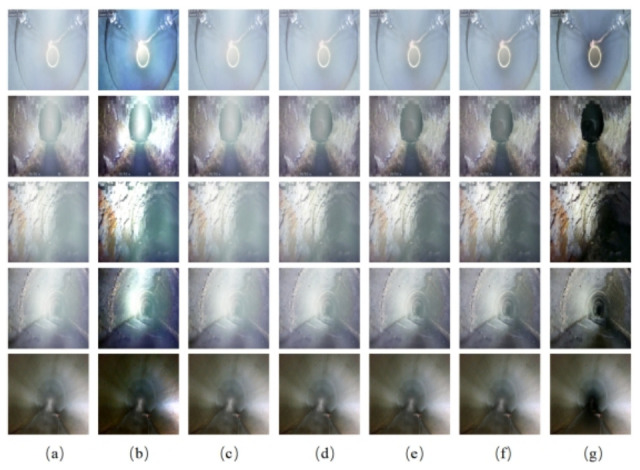
Dehazing results of different algorithms on the drainage pipeline dataset: (**a**) Hazy image, (**b**) gUNet, (**c**) MIMO-UNet, (**d**) MIMO-UNet+, (**e**) MSBDN-RDFF, (**f**) Ours, (**g**) Haze-free image.

The visual dehazing performance of different methods on the drainage pipeline dataset is illustrated in Figure 6. Visual comparisons demonstrate that our proposed MS-PANet exhibits superior performance when processing hazy scenes. This algorithm can generate clearer, more natural haze-free images while preserving the fine details and texture information in the image. Moreover, it effectively avoids image artifacts and color distortion during the dehazing process.

## 5. Conclusions

In this paper, we propose a enhancement method based on a multi-scale spatial pyramid attention (MSPA) mechanism, aimed at addressing the limitations in feature representation and insufficient cross-scale dependency modeling in convolutional neural networks. This module significantly improves the model’s adaptability to complex visual patterns while maintaining computational efficiency through hierarchical feature fusion and structural awareness mechanisms. By incorporating the MSPA module into ResNet, we develop a novel dehazing framework, MS-PANet, that enables adaptive channel attention recalibration while capturing comprehensive multi-scale feature hierarchies. To evaluate dehazing methods within drainage pipeline scenarios, we have also established a benchmark dataset named the CDPD-55000 dataset, which serves as a valuable resource for dehazing algorithm development and evaluation. Our synthetic dataset covers a certain range of scattering coefficients (0.6–1.5) and atmospheric light values to approximate the real pipeline environment, factors such as non-uniform illumination caused by point light sources, turbidity, and anisotropic scattering in real pipelines have not been fully simulated. We acknowledge this gap and consider it as an important direction for future work. Extensive experimental results demonstrate that our method outperforms other approaches in drainage pipeline scenarios, validating the superiority of MSPA. Future work will focus on designing hybrid attention frameworks and exploring dynamic receptive field adjustments to further optimize the trade-off between performance and computational overhead. In future work, we plan to conduct quantitative experiments on how our MS-PANet improves the performance of YOLO-based defect detectors in real pipeline inspection scenarios, thereby directly validating the downstream benefits of image dehazing.

## Figures and Tables

**Table 1:** Comparison of experimental results with different configurations of our MSPA.

Method	PSNR	SSIM
HPC	35.02	0.905
SPR	34.80	0.906
HPC + SPR (no Softmax)	33.97	0.906
HPC + SPR (Softmax)	39.32	0.993

**Table 2:** Performance comparison of MS-PANet on the dataset under different scale parameters *s*. The best results are highlighted in **bold**.

Method	Setting	PSNR	SSIM
MSBDN-RDFF [8]	N/A	33.60	0.905
**MS-PANet (Ours)**	ω=30, s=2	38.27	0.973
	** ω=30, s=3 **	**39.32**	**0.993**
	ω=30, s=4	38.58	0.982
	ω=30, s=5	38.49	0.979

**Table 3:** Performance comparison of MS-PANet on the dataset under different channel parameters ω. The best results are highlighted in **bold**.

Method	Setting	PSNR	SSIM
MSBDN-RDFF [8]	N/A	33.60	0.905
**MS-PANet (Ours)**	ω=28, s=3	38.21	0.972
	** ω=30, s=3 **	**39.32**	**0.993**
	ω=32, s=3	39.34	0.993
	ω=34, s=3	39.35	0.993
	ω=36, s=3	39.37	0.994

**Table 4:** Comparison of experimental results. The best value in each metric is denoted in **bold**.

Method	PSNR	SSIM	Param (M)	Latency (ms)
gUNet [31]	29.33	0.983	5.0	5.38
MIMO-UNet [32]	28.70	0.972	6.8	6.36
MIMO-UNet++ [32]	29.71	0.977	16.1	13.57
MSBDN-RDFF [8]	33.60	0.905	46.35	18.25
DehazeFormer [16]	34.85	0.988	25.44	16.81
MixDehazeNet [33]	35.16	0.906	12.42	12.56
**MS-PANet (Ours)**	**39.32**	**0.993**	**52.8**	**21.28**

## Data Availability

The data presented in this study are openly available in MS-PANet at https://github.com/celicvml/MS-PANet (accessed on 23 April 2026).
